# Supplementary material for: Oncologic Outcomes of Laparoscopic Radical Hysterectomy Using the No-Look No-Touch Technique for Early Stage Cervical Cancer: A Propensity Score-Adjusted Analysis
Source: Cancers (Basel). 2021 Dec 3;13(23):6097. doi: 10.3390/cancers13236097 (PMC8657300; doi:10.3390/cancers13236097)
Supplement: Supplementary file 1 [file cancers-13-06097-s001.zip › cancers-1425712-supplementary.pdf]

cancers

MDPI

Oncologic Outcomes of Laparoscopic Radical Hysterectomy Using the No-Look No-Touch Technique for Early Stage Cervical Cancer: A Propensity Score-Adjusted Analysis

Atsushi Fusegi, Hiroyuki Kanao, Naoki Ishizuka, Hidetaka Nomura, Yuji Tanaka, Makiko Omi, Yoichi Aoki, Tomoko Kurita, Mayu Yunokawa, Kohei Omatsu, Koji Matsuo and Naoyuki Miyasaka

Table S1. Clinicopathological characteristics of patients with tumor size ≥2 cm

	ARH (*n* = 81)	NLNT (*n* = 56)	*P*-value
Age (years)	47.0 (41.0–60.0)	42.0 (36.8–47.3)	0.004
BMI (kg/m^2)	21.5 (20.2–23.9)	20.7 (19.3–23.6)	0.073
Tumor size (cm)	2.8 (2.4–3.5)	2.9 (2.3–3.5)	0.859
Vaginal tumor extension			0.010
Negative	69 (85.2%)	53 (94.6%)	
Positive	12 (14.8%)	3 (5.4%)	
Histological subtype			0.410
SCC	38 (46.9%)	30 (53.6%)	
AC	22 (27.2%)	17 (30.4%)	
ASC	21 (25.9%)	9 (16.0%)	
Post-conization			0.999
No	79 (97.5%)	54 (93.1%)	
Yes	2 (2.5%)	4 (6.9%)	
pT			0.231
1b1	51 (63.0%)	39 (69.6%)	
1b2	9 (11.1%)	3 (5.4%)	
2a1	10 (12.3%)	3 (5.4%)	
2a2	2 (2.5%)	5 (8.9%)	

2b	9 (11.1%)	6 (10.7%)	
pN			0.845
N0	59 (72.8%)	42 (75.0%)	
N1	22 (27.2%)	14 (25.0%)	
Stromal invasion			0.013
<1/2	25 (30.9%)	30 (53.6%)	
≥1/2	56 (69.1%)	26 (46.4%)	
Parametrium invasion			0.999
Negative	72 (88.9%)	50 (89.3%)	
Positive	9 (11.1%)	6 (10.7%)	
Venous invasion			0.587
Negative	54 (66.7%)	34 (60.7%)	
Positive	27 (33.3%)	22 (39.3%)	
Lymphatic invasion			0.730
Negative	39 (48.1%)	29 (51.8%)	
Positive	42 (51.9%)	27 (48.2%)	
Cut margin			0.645
Negative	78 (96.3%)	55 (98.2%)	
Positive	3 (3.7%)	1 (1.8%)	
Adjuvant therapy			0.082
None	26 (32.1%)	22 (39.3%)	
Radiation	1 (1.2%)	0	
Chemotherapy	44 (54.4%)	33 (58.9%)	
CCRT	10 (12.3%)	1 (1.8%)	

Data are presented as medians (interquartile ranges) or n (%). Abbreviations: ARH, abdominal radical hysterectomy; NLNT, total laparoscopic radical hysterectomy using no-look no-touch technique; BMI, body mass index; SCC, squamous cell carcinoma; AC, adenocarcinoma; ASC, adenosquamous carcinoma; CCRT, concurrent chemoradiation therapy.
